# Supplementary material for: A bioinformatics approach to design minimal biomimetic metal-binding peptides
Source: Commun Chem. 2025 Oct 6;8:296. doi: 10.1038/s42004-025-01702-z (PMC12501389; doi:10.1038/s42004-025-01702-z)
Supplement: Supplementary file 2 — Supplemental Material [file 42004_2025_1702_MOESM2_ESM.pdf]

Supplementary Materials for

A bioinformatics approach to design minimal biomimetic metal-binding peptides

Claudia Spallacci,[1] Marco Chino,[2] Antonio Rosato,[3,4] Ornella Maglio,[2,5] Ping Huang,[1] Luca D'Amario,[1] Angela Lombardi,[2]* Claudia Andreini,[3,4]* Mun Hon Cheah[1]*

Affiliations
[1] Molecular Biomimetics, Department of Chemistry-Ångström Laboratory, Uppsala University, Box 523, 75120, Uppsala, Sweden.
[2] Department of Chemical Sciences, University of Naples Federico II, Via Cintia 21, 80126 Napoli, Italy.
[3] Department of Chemistry, University of Florence, Via della Lastruccia 3, 50019 Sesto Fiorentino, Italy.
[4] Magnetic Resonance Center (CERM), University of Florence, Via L. Sacconi 6, 50019, Sesto Fiorentino, Italy
[5] Institute of Biostructures and Bioimaging, National Research Council, Via P. Castellino, 111, 80131 Napoli, Italy

*Corresponding authors:
Angela Lombardi: alombard@unina.it
Claudia Andreini: andreini@cerm.unifi.it
Mun Hon Cheah: michael.cheah@kemi.uu.se

This PDF file includes:

Figs. S1 to S17

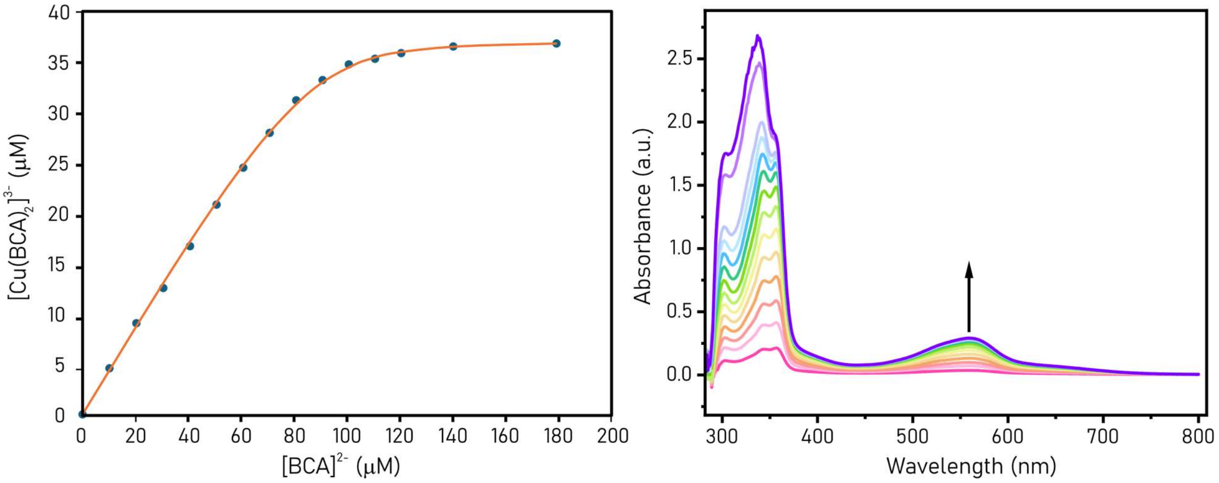

Fig. S1.

Fitting results of competitive binding assay with BCA. Competitive titration experiments were performed using BCA (bicinchoninic acid) as a competing ligand ($\log\beta_2$ = 17.2, 7.2 mM stock solution). Formation of $[Cu(BCA)_2]^{3-}$ was monitored spectrophotometrically at 562 nm (ε_{562} = 7900 M^{-1} cm^{-1}) by adding small aliquots of the BCA stock to a solution containing H4pep-Cu^+ complex (40 μM, prepared with 0.95 equivalents of 10 mM $[Cu(CH_3CN)_4]PF_6$ dissolved in acetonitrile) in 50 mM sodium phosphate buffer at pH 5.8 with 1 mM sodium ascorbate under Argon atmosphere. A dissociation constant of K_D = 2.4*10^{-12} M was estimated by fitting the titration data as described in the Materials and Methods section of the main text.

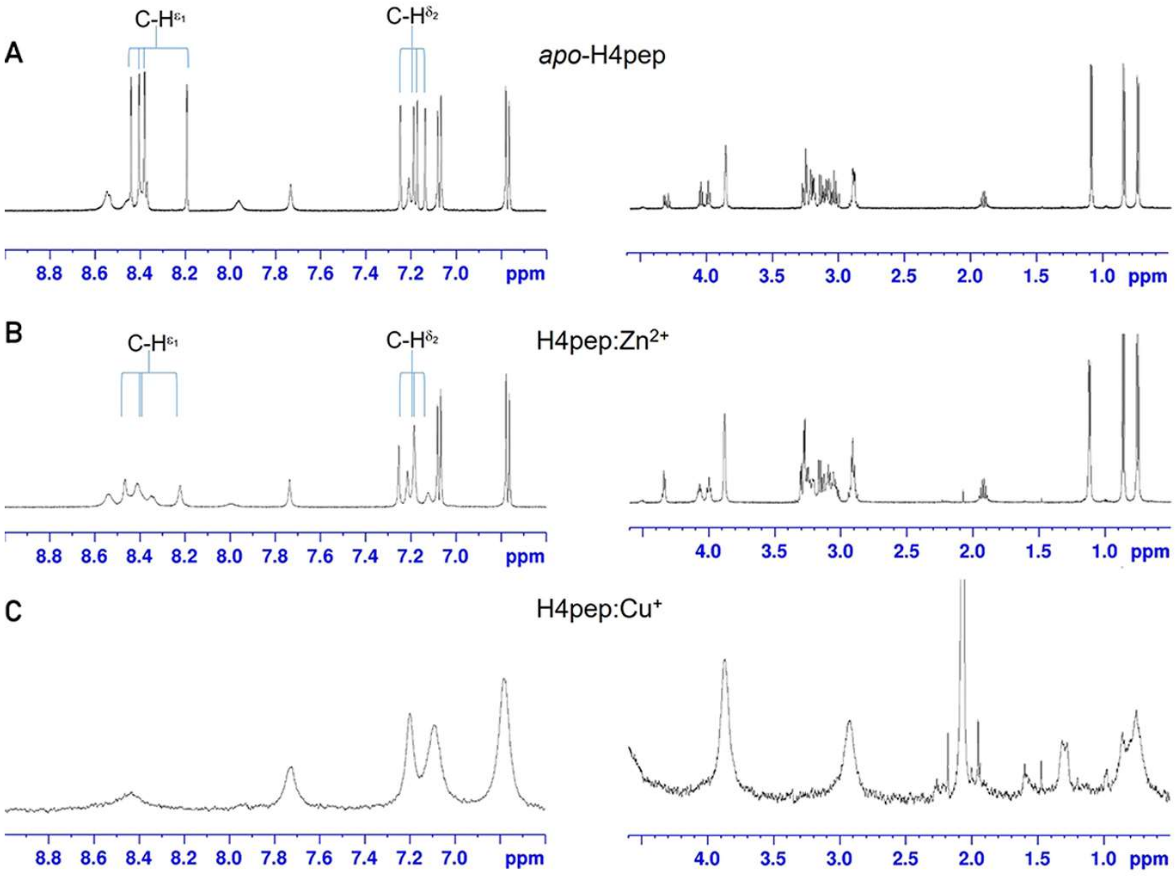

Fig. S2.

NMR spectra of apo and metal-bound H4pep in aqueous solution. (**A**) apo-H4pep, (**B**) 1:1 H4pep:Zn^{2+}, and (**C**) 1:1 H4pep:Cu^{+} NMR spectra recorded in 50 mM sodium phosphate buffer at pH 5.8. Addition of Zn^{2+} and Cu^{+} to H4pep leads to broadening of the histidine proton resonances, indicating that metal binding involves these residues.

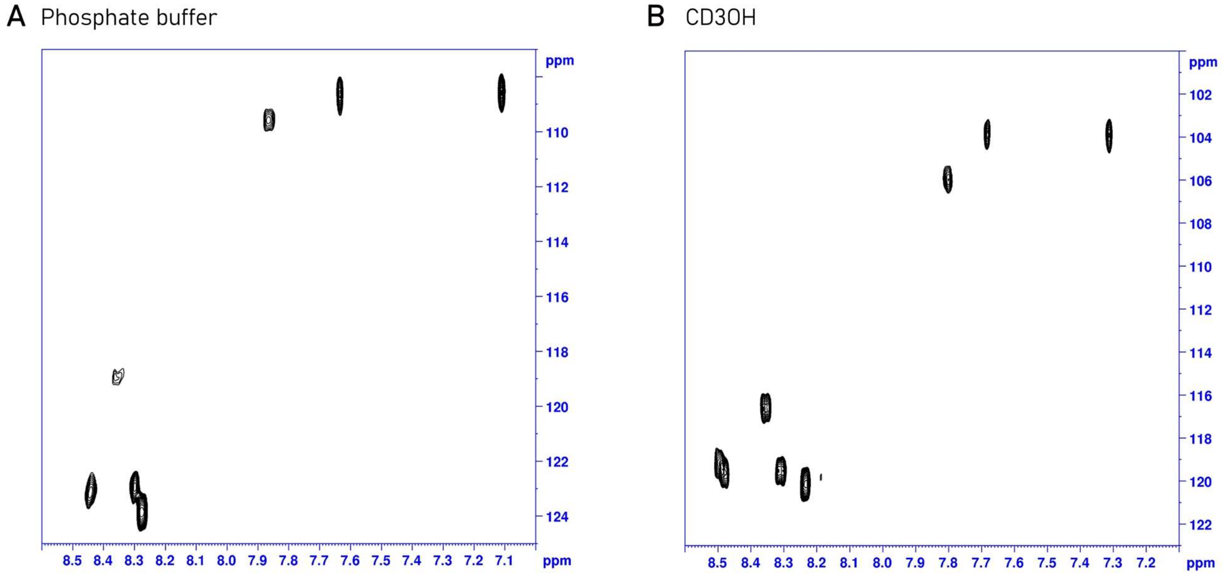

Fig. S3.

2D ^{1}H-^{15}N HSQC spectrum of apo-H4pep. Proton resonances could be assigned to backbone amide protons and to the terminal amide group. Spectra were recorded at natural abundance in sodium phosphate buffer 50 mM at pH 5.8 (**A**) and CD_3OH (**B**).

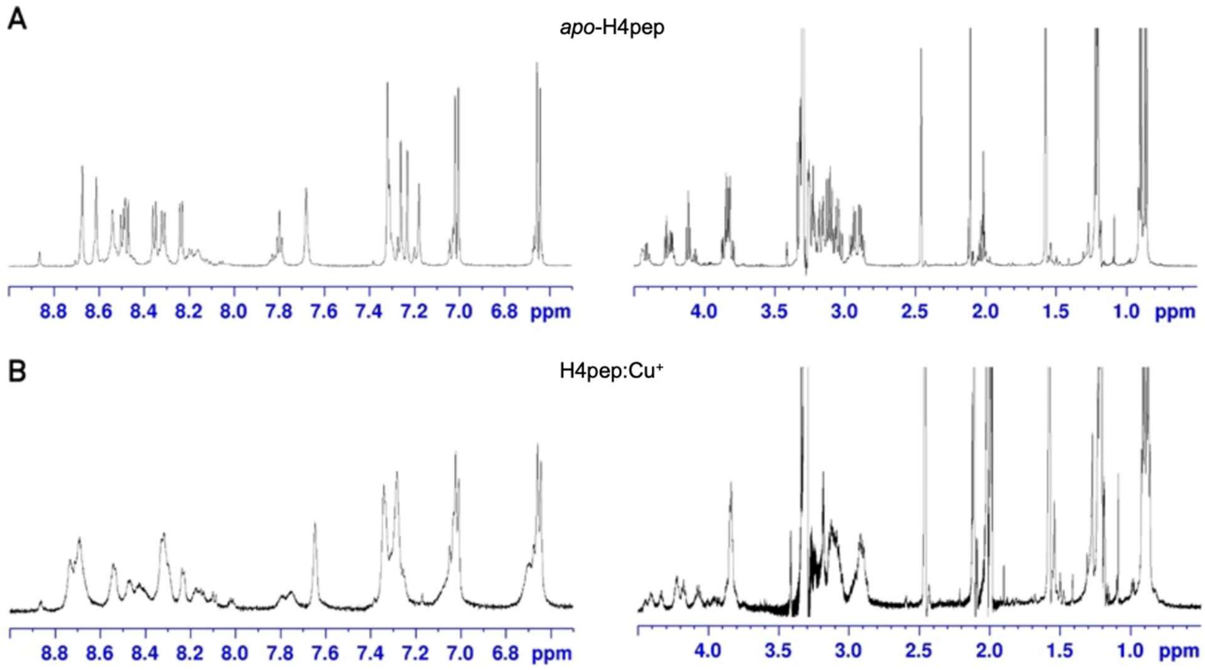

Fig. S4.

NMR spectra of apo and metal-bound H4pep in methanol. (**A**) apo-H4pep, and (**B**) 1:1 H4pep:Cu^+spectra recorded in CD_3OH. The same broadening of histidine proton resonances as observed in aqueous solution confirms their role in coordinating the Cu^+.

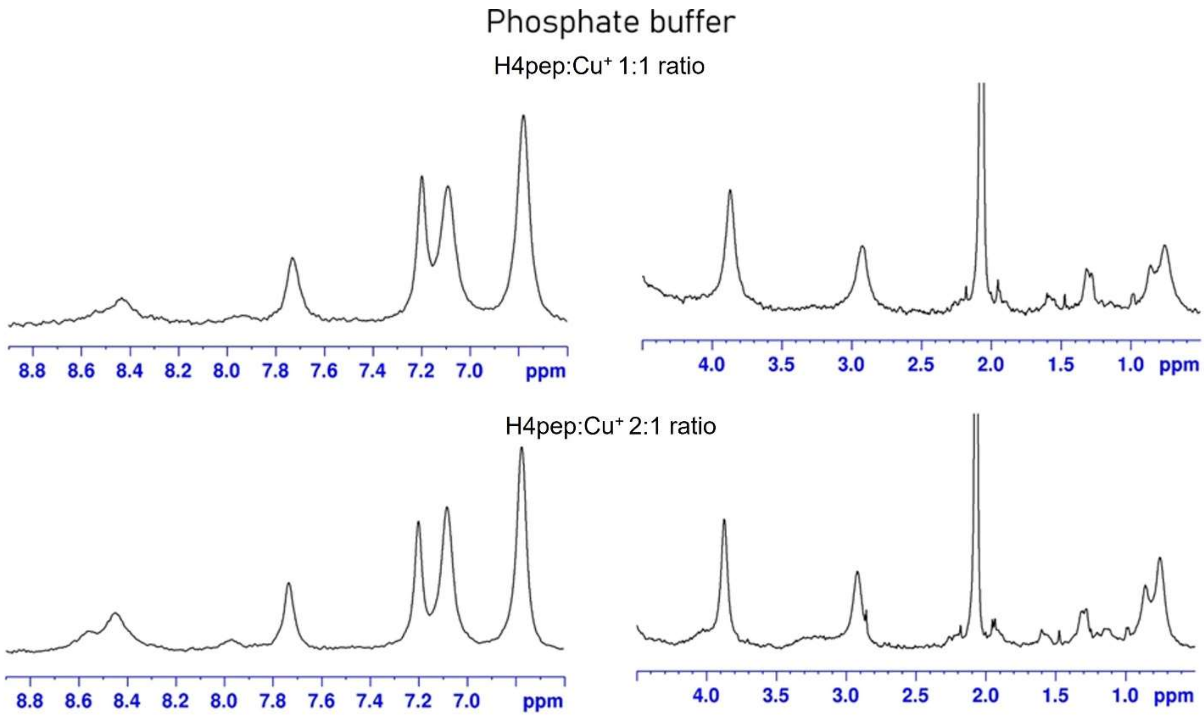

Fig. S5.

NMR spectra of H4pep:Cu^{+} at different ratios in aqueous solution. Spectra of H4pep:Cu^{+}in a 1:1 ratio (top) and in a 2:1 ratio (bottom). No significant differences were observed between the two conditions. The spectra were recorded in 50 mM sodium phosphate buffer at pH 5.8.

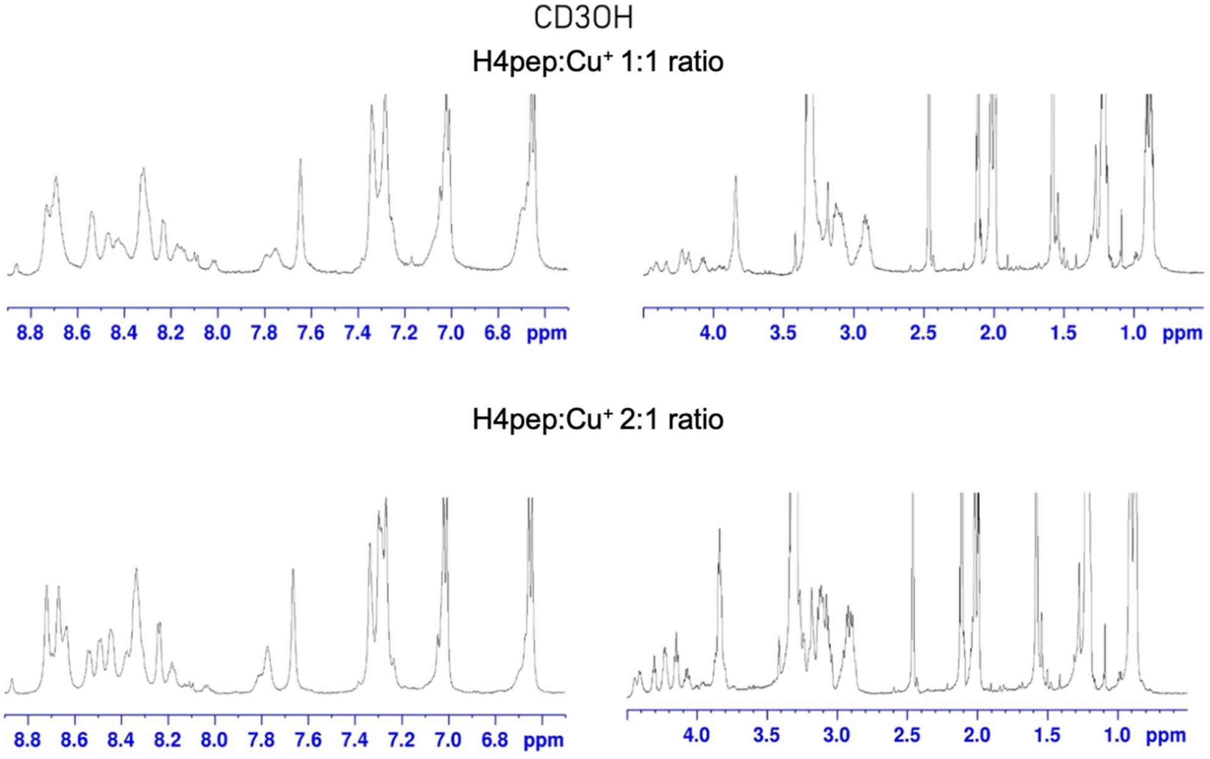

Fig. S6.

NMR spectra of H4pep:Cu^+ at different ratios in methanol. Spectra of H4pep:Cu^+ in a 1:1 ratio (top) and in a 2:1 ratio (bottom). No significant differences were observed between the two conditions.

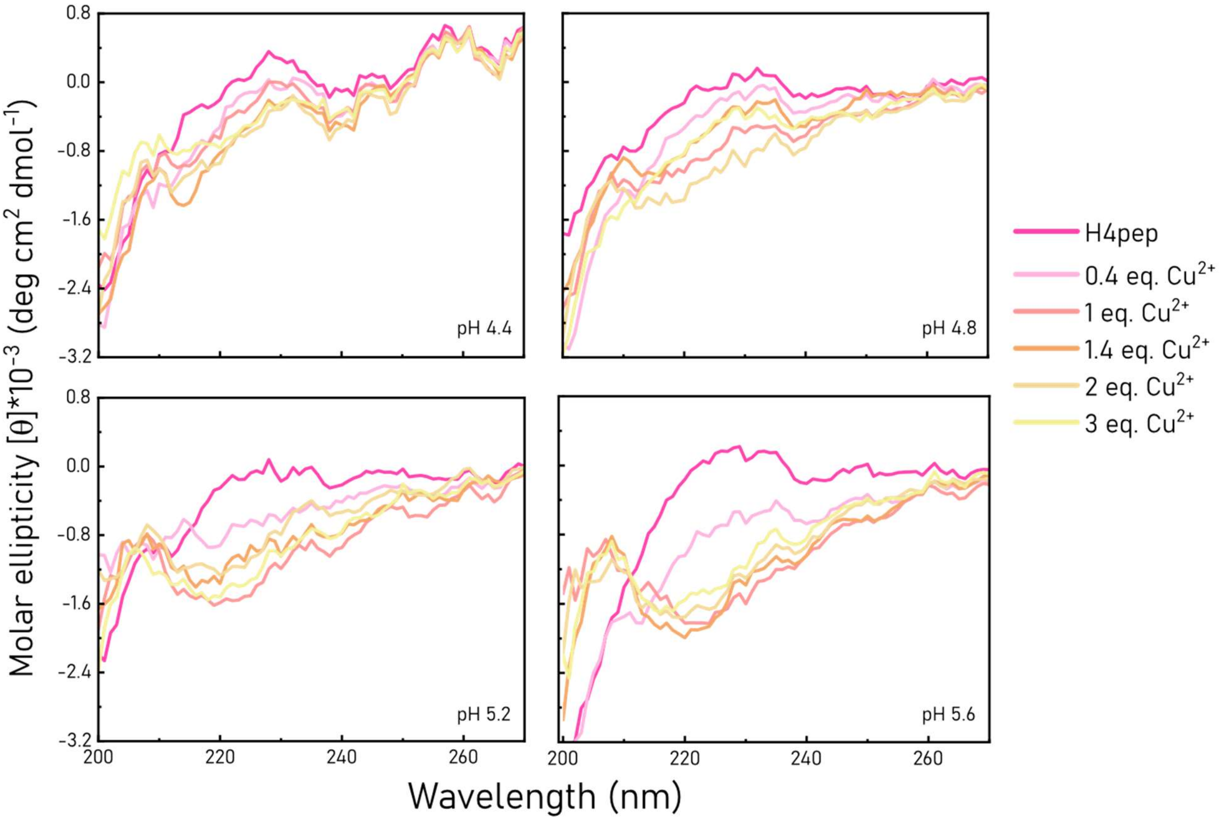

Fig. S7.

CD spectra of H4pep titration with Cu^{2+} at different pHs. Spectra were recorded in 10 mM acetate buffer in presence of a peptide concentration of 0.1 mM. Spectra were smoothed via adjacent averaging of 5 data points. Conformational change from random coil to a more definite secondary structure is observed only starting from pH 5.2.

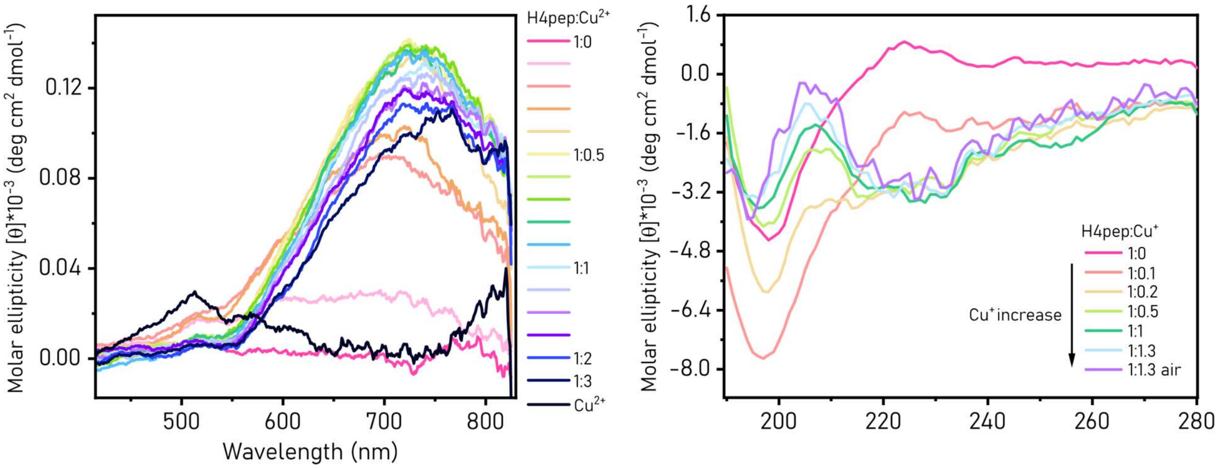

Fig. S8.

CD spectroscopy of H4pep-Cu^{2+} and H4pep-Cu^{+} complexes. (**A**) visible-CD spectra of H4pep titration with Cu^{2+} ([H4pep] = 1 mM, pH 5.6). Spectra were smoothed via adjacent averaging of 30 data points. The rise of a feature in this range of the CD spectrum is consistent with Cu^{2+} binding to the peptide. (**B**) CD spectra of H4pep titration with Cu^{+} ([H4pep] = 0.1 mM, pH 5.6). Spectra were smoothed via adjacent averaging of 5 data points. Binding of Cu^{+} to the peptide seems to lead to a similar conformation to that of H4pep-Cu^{2+}.

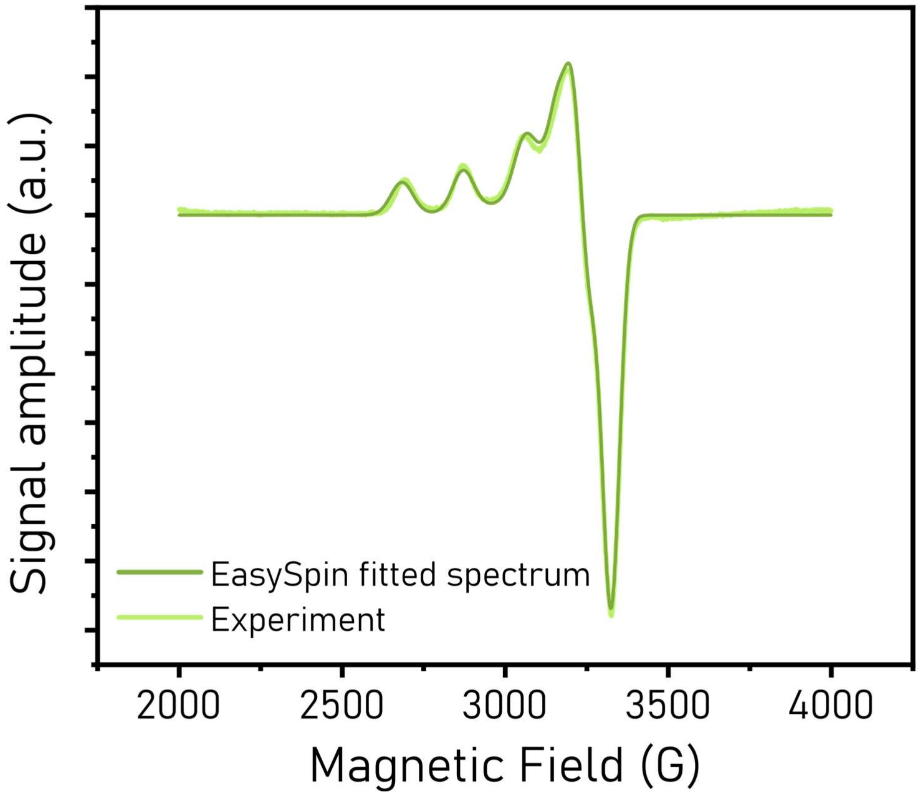

Fig. S9.

Fitted EPR spectra of the 1Cu2Pep species. Results of the fitting: 69% ^{63}Cu 31% ^{65}Cu; A_x = 21.87; A_y = 61,25; A_z = 577.2; g_x = 2.0422; g_y = 2.0732; g_z = 2.2447. The EasySpin software version 6.0.5 was used for spectral simulation and fitting (refs. *56*, *57* in the main text). The spin system used in the program was set as s = ½, for Cu^{2+} possessing merely one unpaired electron (d^9 electronic configuration). In this system, the hyperfine interaction (i.e., electron-nuclear interaction) is clearly demonstrated in the low field region with typically 500 – 600 MHz line splitting. Thus, the hyperfine interaction was included in the spin Hamiltonian. The simulation function "pepper" provided by the EasySpin program, in combination with the command "esfit", was used to fit the experimental data.

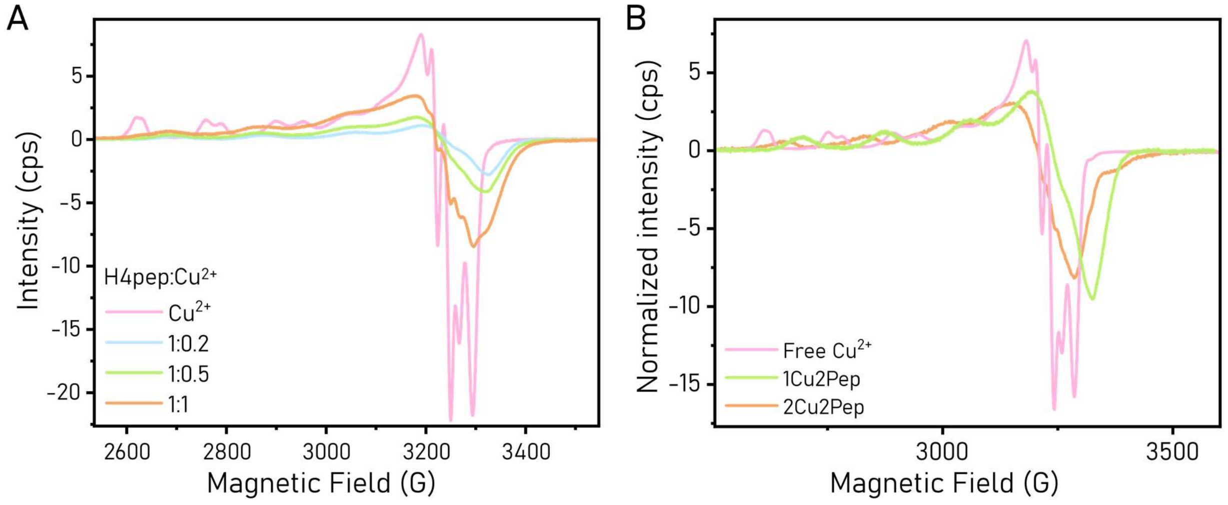

Fig. S10.

EPR spectra of different H4pep-Cu^{2+} species. (**A**) EPR spectra of samples containing a fixed concentration of H4pep (0.1 mM) and increasing equivalents of Cu^{2+}. (**B**) EPR spectra of H4pep-Cu^{2+} isolated species, obtained by spectral deconvolution and assigned according to the "Stoichiometry of H4pep-Cu^{2+} complexes" section. The spectra were normalized based on the double integral of the Cu^{2+} standard spectrum.

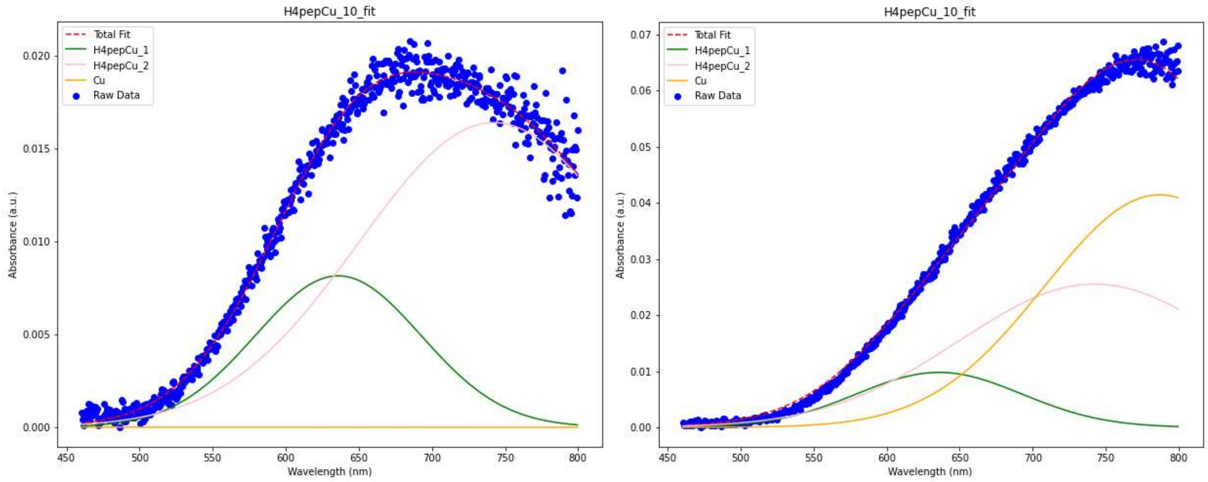

Fig S11.

Deconvolution of UV-visible spectra. Example of fitting of UV-visible band in presence of a H4pep:Cu^{2+} ratio of 1:0.2 (left) and 1:2 (right). Up to 1 Cu equivalents, the bands could be fitted with two Gaussian components (H4pepCu_1 and H4pepCu_2 in the legend); from fitting the H4pep:Cu^{2+} 1:0.2 sample, peak position and width of the Gaussian curves representing the H4pepCu_1 and H4pepCu_2 species were extracted and assumed constant in fitting samples at ratios 1:0.3 to 1. At Cu^{2+} concentrations > 1 equivalent, a third component was included to ascribe for the presence of free Cu^{2+} in solution (orange curve). The position and width of the Gaussian curve representing free Cu^{2+} were extracted from a sample containing only Cu^{2+} ions, and assumed constant in fitting samples at ratios 1:1.1 to 1:2. The amplitudes of the Gaussian curves resulting from the fitting were used to obtain the UV-visible speciation plot reported in Fig. 2D of the main text.

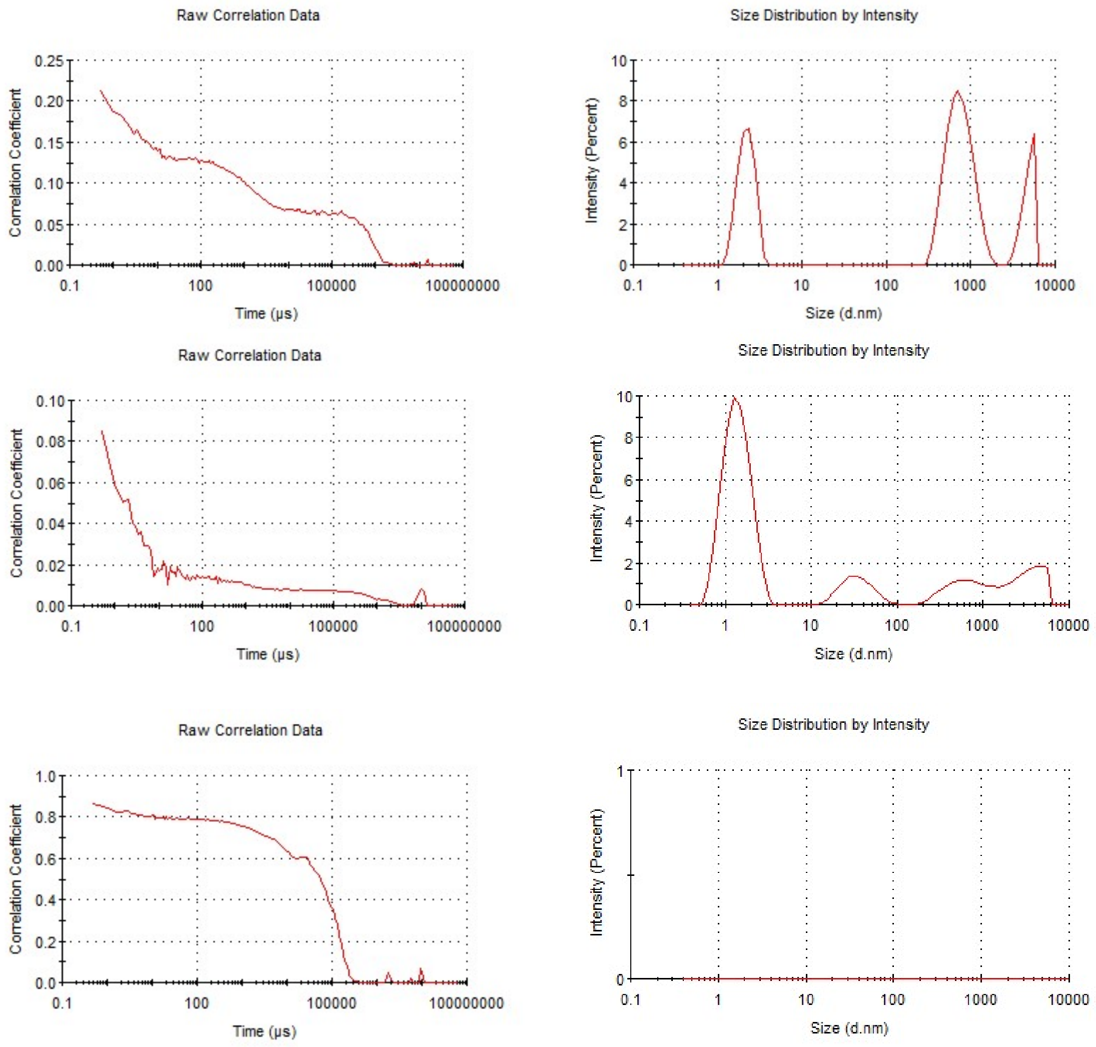

Fig. S12.

DLS measurements of H4pep-Cu^{2+}. Three DLS measurement repeats on the same sample of a 1 mM solution of H4pep with addition of 3 Cu^{2+} equivalents show inconsistent results, confirming the absence of large aggregates of definite size. Background noise is detected.

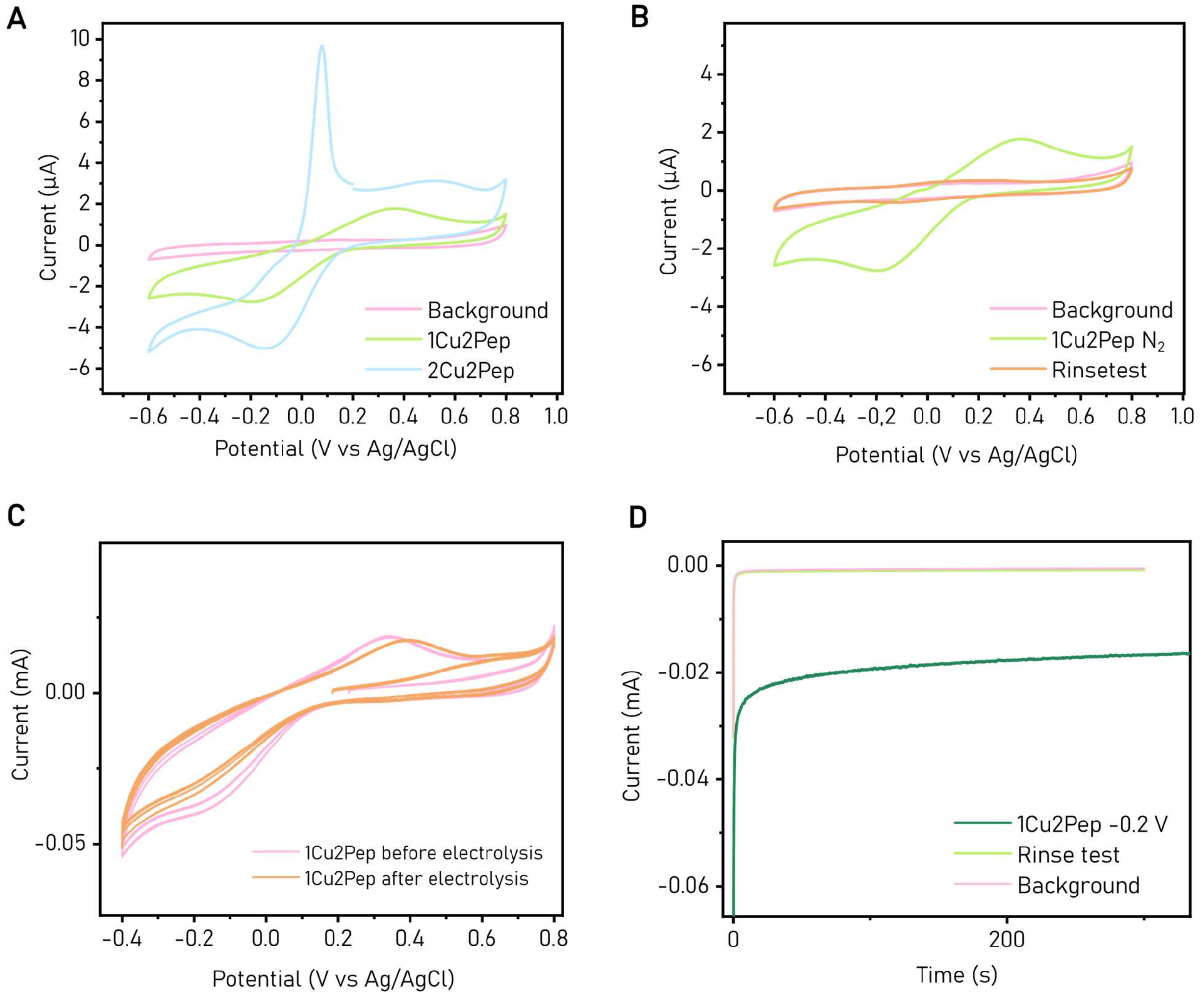

Fig. S13.

Electrochemistry of 1Cu2Pep and 2Cu2Pep species. (**A**) CV traces recorded in presence of 1 mM H4pep and 0.45 mM Cu^{2+} (1Cu2Pep, green trace) or 0.9 mM Cu^{2+} (2Cu2Pep, blue trace). CV of 2Cu2Pep shows a sharp oxidation peak at 0.08 V, characteristic of oxidation of metallic Cu deposited on the working electrode. On the other hand, such feature is not observed in the CV of **1Cu2Pep**, which shows a reduction peak at -0.16 V and the corresponding reoxidation peak at 0.36 V. (**B**) Rinse test performed after CV of the 1Cu2Pep species is comparable to the background, demonstrating no complex is deposited on the working electrode. The CV traces in A and B were recorded in 10 mM sodium phosphate buffer and 40 mM Na_2SO_4 supporting electrolyte. The solution was bubbled with N_2 gas for five minutes just before the measurement. A 3 mm glassy carbon plate was used as working electrode. (**C**) CV traces of 1Cu2Pep before and after bulk electrolysis at -0.2 V show no significant differences, highlighting the stability of the complex. (**D**) Rinse test performed after bulk electrolysis at -0.2 V of 1Cu2Pep (light green trace) displays comparable current to the background (pink trace), showing no active species are deposited on the working electrode. The traces in C, D and E were recorded in 10 mM sodium phosphate buffer and 40 mM Na_2SO_4 supporting electrolyte. A 9 mm glassy carbon plate was

used as working electrode. All potentials are expressed versus a Ag/AgCl reference electrode ($E°_{Ag/AgCl}$ = + 0.210 V vs SHE).

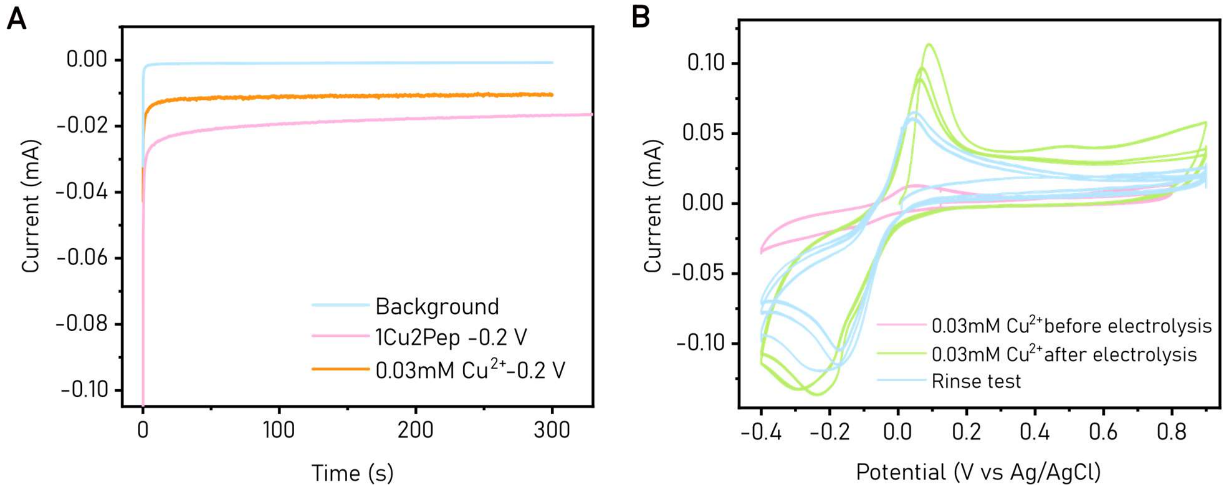

Fig. S14.

Electrochemical behavior of free Cu^{2+} compared to 1Cu2Pep. (**A**) Bulk electrolysis performed at -0.2 V of only a small concentration of free Cu^{2+} (0.03 mM, orange trace) shows a lower reductive current compared to 1Cu2Pep ($[Cu^{2+}]$ = 0.045 mM). (**B**) A sharp oxidation peak around 0.1 V of the sample containing 0.03 mM free Cu^{2+} is visible in the CV traces recorded after electrolysis and in the rinse test. This is indicative of deposition of Cu^0 species on the working electrode surface.

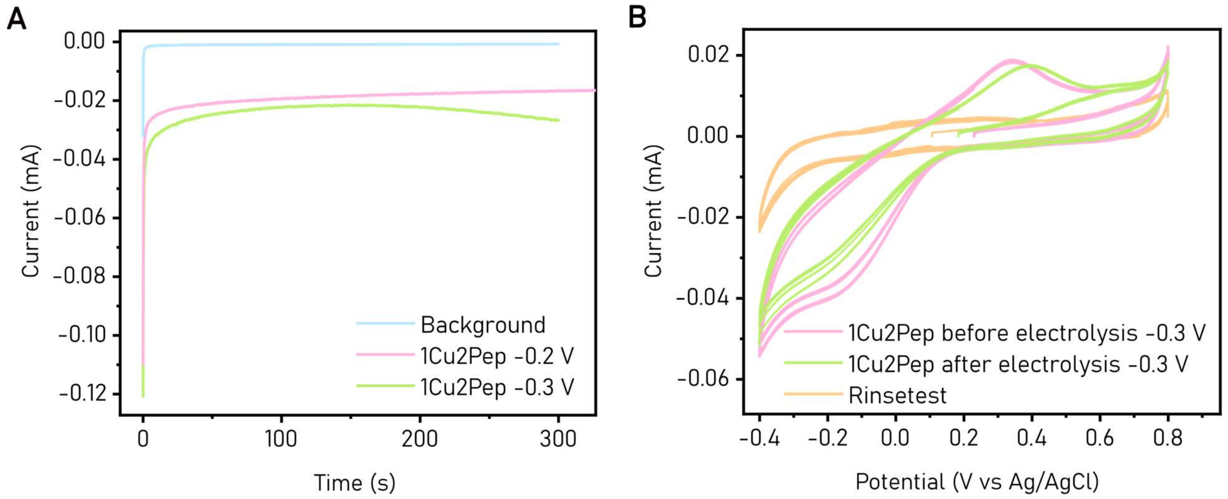

Fig. S15.

Electrochemical behavior of 1Cu2Pep at more negative reducing potential. (**A**) Bulk electrolysis of 1Cu2Pep at -0.3 V (green trace) shows an initial decrease in reducing current, followed by a gradual increase after 150 seconds. This suggests that a more active species of the H4pep:Cu assembly can be formed in these conditions. (**B**) CV traces of the same sample before and after electrolysis show no significant differences, suggesting that the complex remained stable during the measurement.

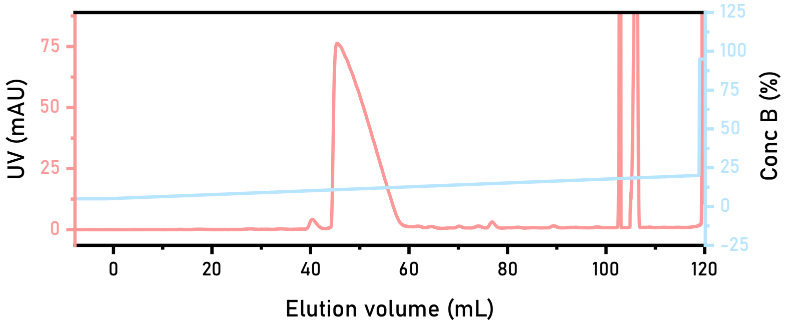

Fig. S16.

HPLC chromatogram of H4pep. The peptide was purified over a C18 semi-preparative column and eluted at 10% solvent B (0.085% TFA in acetonitrile).

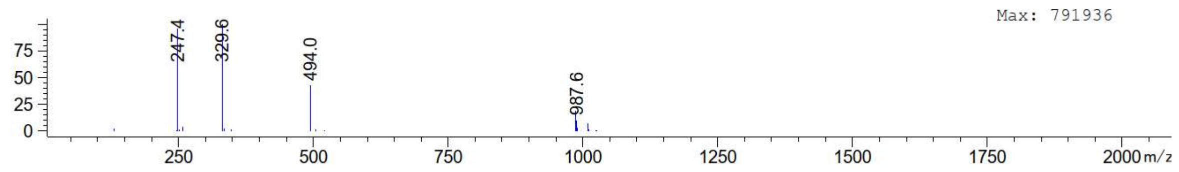

Fig. S17.
Mass spectra of H4pep. The molecular weight of the purified peptide was confirmed via LCMS ($[M+H]^+$ = 987.6 m/z).
