# Supplementary material for: A bioinformatics approach to design minimal biomimetic metal-binding peptides
Source: Commun Chem. 2025 Oct 6;8:296. doi: 10.1038/s42004-025-01702-z (PMC12501389; doi:10.1038/s42004-025-01702-z)
Supplement: Supplementary file 3 — Description of Additional Supplementary Files [file 42004_2025_1702_MOESM3_ESM.pdf]

Description of Additional Supplementary Files

File name: Supplementary Data 1

Description: Coordinates of 1Cu2Pep from DFT optimization

File name: Supplementary Data 2

Description: Coordinates of 2Cu2Pep from DFT optimization

File name: Supplementary Data 3

Description: NMR dataset
